# Effects of intranasal dantrolene nanoparticles on brain concentration and behavior in PS19 tau transgenic mice

**DOI:** 10.21203/rs.3.rs-2802620/v1

**Published:** 2023-05-11

**Authors:** Robert Vera, Nicholas Hong, Bailin Jiang, Grace Liang, Maryellen F Eckenhoff, Halle J Kincaid, Veron Browne, Vinolia Chellaraj, Douglas Gisewhite, Michael Greenberg, Sudhir Ranjan, Gaozhong Zhu, Huafeng Wei

**Affiliations:** University of Pennsylvania, Perelman School of Medicine; University of Pennsylvania, Perelman School of Medicine; Peking University People’s Hospital; University of Pennsylvania, Perelman School of Medicine Maryellen; University of Pennsylvania, Perelman School of Medicine; University of Pennsylvania, Perelman School of Medicine; Eagle Pharmaceuticals, Inc; Eagle Pharmaceuticals, Inc; Eagle Pharmaceuticals, Inc; Eagle Pharmaceuticals, Inc; Eagle Pharmaceuticals, Inc; Eagle Pharmaceuticals, Inc; University of Pennsylvania, Perelman School of Medicine

**Keywords:** calcium, dantrolene, ryanodine receptor calcium release channel, blood-brain barrier, pharmacokinetics, Alzheimer’s disease, PS19 mice, tau protein, cognition, therapeutics

## Abstract

**Background:**

Repurposing dantrolene as a potential disease-modifying treatment for Alzheimer’s disease has been shown to be effective in amyloid transgenic mouse models but has not been examined in a model of tauopathy.

**Objective:**

The effects of a nanoparticle intranasal formulation, the Eagle Research Formulation of Ryanodex (ERFR), in young adult and aged wild type and PS19 tau transgenic mice was investigated.

**Methods:**

The bioavailability of intranasal ERFR was measured in 2 months and 9–12 month old C57BL/6J male mice. Mice received a single intranasal dose of ERFR and, after 20 min, blood and brain samples were collected. Dantrolene concentrations in the plasma and brain were analyzed by High Performance Liquid Chromatography. Animal behavior was examined in PS19 tau transgenic mice, with/without acrolein treatment to exacerbate cognitive deficits. Behavioral tests included cognition (cued and contextual fear conditioning, y-maze), motor function (rotarod), and olfaction (buried food test).

**Results:**

Dantrolene concentration in the blood and brain decreased with age, though the decrease was greater in the blood resulting in a higher brain to blood concentration ratio. The behavioral assays showed no significant changes in cognition, olfaction or motor function in the PS19 mice compared to controls after chronic ERFR treatment even with acrolein treatment.

**Conclusion:**

Our studies suggest that while we did not find PS19 mice to be a reliable Alzheimer animal model to test the therapeutic efficacy of dantrolene, the results suggest a potential for ERFR to be an effective chronic therapy for Alzheimer’s disease and that further studies are indicated.

## INTRODUCTION

Over 95% of AD patients suffer from sporadic AD (SAD), which is typically late-onset AD (LOAD) in people over 65 years of age, while only about 5% of AD patients present with familial AD (FAD), which usually occurs decades earlier[1]. Despite the tremendous international effort to develop drugs for the treatment of AD, no disease-modifying drugs are available currently. One of the proposed strategies for the development of new AD drug treatments is targeting the primary upstream AD pathology in the brain, such as cellular calcium (Ca^2+^) dysregulation.

Dantrolene is the primary and only FDA-approved treatment for malignant hyperthermia, a severe reaction to anesthesia caused by over activation of type 1 ryanodine receptor (RyR-1) Ca^2+^ channels located on the membrane of the sarcoplasmic reticulum (SR) in muscle cells[2]. This over activation of the RyRs receptors, leading to Ca^2+^ dysregulation, is similar to what is found in the brain with Alzheimer’s disease. Increasing evidence suggests that dantrolene could be neuroprotective against neurodegenerative diseases including Alzheimer’s disease [3–8], Huntington’s disease[9], cerebral ischemia[10–12], spinal cord injury[13, 14], and seizures[15, 16], by its ability to inhibit the over activation of ryanodine receptors (RyRs) in the central nervous system (CNS).

Calcium dysregulation in AD, due to the over activation of ryanodine receptor (RyRs) Ca^2+^ channels located on the membrane of the endoplasmic reticulum (ER) in neurons, results in excessive Ca^2+^ release from the ER, leading to a pathological elevation of Ca^2+^ concentration in the cytosol ([Ca^2+^]_c_) and mitochondria ([Ca^2+^]_m_), as well as depletion of the Ca^2+^ concentration in the ER ([Ca^2+^]_er_)[17]. This Ca^2+^ dysregulation in AD leads to downstream pathologies such as amyloid/tau pathology, mitochondrial dysfunction, generation of reactive oxygen species (ROS), inflammation, apoptosis, and impaired autophagy, neurogenesis, and synaptogenesis [4, 18–20]. Therefore, an effective therapeutic approach for AD would be restoring Ca^2+^ homeostasis by inhibiting the over activation of RyRs [4].

Dantrolene has been demonstrated to be neuroprotective in AD models, including cell culture [7, 21] and FAD animal models[3, 5, 6, 8, 22]. Although dantrolene is a small lipid-soluble molecule, it has a limited ability to pass through the blood-brain barrier (BBB) and enter the CNS, making it a less effective treatment via intravenous or oral administration. However, the BBB becomes increasingly disrupted with aging and favors drug penetration into the CNS in AD patients, especially with late onset disease [1].

The effects of aging on intranasal dantrolene penetration into the brain compared to blood levels needs to be investigated in animals. Our previous studies have demonstrated that the intranasal administration of dantrolene in a nanoparticle solution significantly increased its brain concentration and brain/blood concentration ratio, compared to oral or subcutaneous injection of dantrolene in 2 months old wild type mice[6, 23]. The chronic intranasal administration of dantrolene abolished the memory learning and memory impairment in 5XFAD mice, an aggressive FAD mouse model, even when treatment was initiated after the onset of AD pathologies and cognitive dysfunction[6]. The effects of dantrolene on amyloid plaque levels in AD mouse models has been inconsistent, with no change in 5XFAD[6] mice, increase[24] and decreased amyloid levels in different amyloid transgenic AD models [3, 22]. The effects of dantrolene on tau pathology, another hallmark of AD, and cognition have not been studied in animal models.

In this study, we investigated the effects of aging on brain versus blood dantrolene concentrations after intranasal administration of an Eagle Research Formulation of Ryanodex (ERFR), a dantrolene nanoparticle formulation. We also studied the effects of this ERFR on cognitive function in commonly used tau PS19 transgenic mice[25, 26].

## MATERIALS AND METHODS

### Animals and treatment groups

All procedures were carried out in accordance with the ARRIVE guidelines and protocols approved by the Institutional Animal Care and Use Committee (IACUC) at the University of Pennsylvania. Male and female mice, including C57BL/6J mice (Strain #000664) at 2 and 9–11 months of age, and tau point mutation transgenic mice (P301S, Line PS19) and non-transgenic littermates at 6 and 9 months of age, weighing 29–44g, were used in these experiments. Mice were maintained in the University of Pennsylvania laboratory animal research housing at 21–22°C with a 12-hour light-dark cycle and provided with food and water ad libitum. Body weights were monitored every week. All efforts were made to minimize the suffering and number of mice according to IACUC guidelines.

For the brain/blood concentration study, the intranasal Eagle Research Formulation of Ryanodex (ERFR) was administered one time to C57BL/6 mice (WT) at 2 months old (N = 10) and 9–12 months old (N = 9). For the behavioral studies, Tau PS19 transgenic mice and non-transgenic littermates (NT) underwent behavioral assays to determine baseline cognition, olfaction, and motor function. Another cohort of mice, beginning at 2 months of age, were given the intranasal ERFR daily for 3 months, to determine the effect of ERFR on cognitive function. Acrolein was also administered together with ERFR to increase cognitive dysfunction in this tau mouse model. PS19 and WT mice were given either vehicle (water), acrolein respectively, ERFR, or ERFR plus acrolein respectively.

### Intranasal administration of dantrolene

Intranasal administration of dantrolene was performed using a method we have described previously[6, 7]. Briefly, the ERFR was provided by Eagle Pharmaceuticals, Inc. Aliquots were stored at −80°C and thawed at room temperature for use. ERFR was shielded from light using aluminum-wrapped vials and pipette tips. Animals were weighed before each dosing. A P200 analytical pipette with a standard 2–200 μL pipette tip was used to deliver 1 μL per gram of body weight. The mouse was held with one hand in a suspended supine position. Small droplets of ERFR were dripped very slowly into the nose until fully absorbed, with a 2–3 second break period between each droplet delivery. Each mouse was held still for 10–15 seconds after dosing completion. Total administration time per mouse was between 1–3 minutes. Mice for the brain/plasma concentration study were given 1 dose and mice for the behavioral studies were given 1 dose/day, 5 days/ week, for 3 consecutive months, starting at 2 months old. Acrolein (2 mg/kg) was administered by gavage fed daily for 5 days per week for three months[27].

### Dantrolene brain/blood concentration ratio study

For the brain and blood concentration study, 20 minutes after the completion of each intranasal administration of ERFR, each animal was individually anesthetized in a 2–4% isoflurane in 30% oxygen carrying gas chamber until breathing rate reached approximately 50% of normal rate. The animal was removed from the chamber and placed in a supine position on the operating platform in a fume hood. An isoflurane nose cone was used to maintain a surgical plane of anesthesia as determined by lack of response to a toe pinch. Isoflurane concentration was kept at 1–2% and was adjusted as necessary.

Prior to the euthanasia procedure, two 1-inch, 22G needles were attached to 3 mL syringes and washed with 1000 IU/mL heparin for blood collection. A 30G needle was attached to a peristaltic pump, and the intake tube was placed in a 0.9% saline/heparin (3 IU/mL) solution. The skin over the chest cavity was removed and the chest cavity opened to expose the chest cavity and heart. A syringe was inserted into the left ventricle to collect arterial blood into heparin-washed microfuge tubes. Additional blood in the chest cavity was collected with a syringe into heparin-washed tubes. Anticoagulated blood samples were kept on ice until the animal was euthanized. The samples were then centrifuged at 2500 G for 15 minutes at 4°C and the plasma supernatant was collected and transferred to Micronics vials for storage at −80°C.

After the blood collection, while the animal was still under anesthesia, the animal was euthanized by inserting the pump needle into the left ventricle, the right atrium was cut, and cardiac perfusion and exsanguination was performed with a saline/heparin solution for 30 seconds at 10 mL/min. The brain was removed, weighed, rapidly frozen in liquid nitrogen then stored at −80°C. All brain and plasma samples were subsequently shielded from light until assayed.

### Bioanalytical Method

Brain and plasma samples were quantified by LC-MS/MS method. The bioanalytical method involves extraction of Dantrolene and 5-OH-Dantrolene from mice plasma (K2EDTA and brain samples homogenized in 70% Isopropyl alcohol in water) prior to extraction. The samples were extracted by protein precipitation using acetonitrile, containing the internal standard (Dantrolene-d4 and 5-OH- Dantrolene-d4). The analytes and internal standards are separated using a reversed phase liquid chromatography gradient and quantified with tandem mass spectrometric detection. The range of quantification for the method is 10ng/mL – 1000 ng/mL for plasma method and 1ng/mL-1000ng/mL for the brain method. The analysis was performed on a triple-quadrupole LC-MS/MS (AB Sciex API 5500 QQQ) with Sciex ExionLC UPLC (ExionLC AD Pump) with Sciex ExionLC AD Multiplate Sampler and Sciex ExionLC AC Column Oven. The autosampler temperature was set at 4–10°C. Liquid chromatographic separation was performed on Restex Force Biphenyl (2.1 × 30mm, 1.8 μm) and the temperature was set at 40°C. The mobile phase had a flow rate of 0.4 mL/min and followed an elution gradient program using Mobile phase A (10 mM Ammonium Acetate + 0.1% Acetic Acid in Water) and Mobile Phase B (10 mM Ammonium Acetate + 0.1% Acetic Acid in 90:10 ACN:Water). Peak area ratios of the analytes and the IS were used to calculate concentrations. The MS was operating in a negative electrospray ionization mode and multiple reaction monitoring (SRM) mode with a negative spray voltage of −4500V.

### Behavioral Studies

Tau PS19 transgenic mice and non-transgenic littermates, at 6 and 9 months of age, underwent several behavioral paradigms to evaluate learning and memory in the absence of intranasal ERFR. Another cohort of Tau PS19 transgenic mice and non-transgenic littermates at age of 2 months old received intranasal ERFR, with or without acrolein. The investigator was blinded to the experimental groups.

### Fear Conditioning test

The contextual and cued fear conditioning test is a behavioral paradigm used to assess associative fear, hippocampal dependent and independent, learning and memory in mice, as we have described previously[6]. On the first day, mice were acclimated to the testing environment for 1 h. The animals were placed in a conditioning chamber and given 3 pairings of a conditioned stimulus (auditory cue) and an aversive unconditioned stimulus (electric foot shock). On the second day, for the contextual fear conditioning test (hippocampal dependent memory), the mice were exposed to the same conditioning chamber without the auditory cue or foot shock and freezing time recorded. Two hours later, for the cued fear conditioning test (hippocampal independent memory), the mice were placed in a different chamber with no auditory cue or foot shock for the first 3 min, followed by 3 cycles of the previous auditory cue and freezing time recorded[6]. Freezing behavior during the tests was measured as an index of fear memory and were recorded using the ANY-maze Fear Conditioning System (Model: 46000–590, UGO Basile, Gemonio Italy) equipped with a video camera and ANY-maze software (V.4.99 Stoelting Co. Wood Dale, IL).

### Y-Maze Spontaneous Alternation Behavior

Y maze testing is used to measure short term spatial working memory. The test was carried out using a Y-shaped maze with three light-colored, opaque arms orientated at 120° angles from each other, with a method described previously with some modification[28]. Briefly, the mouse was introduced at a particular position on the maze and allowed to explore the arms freely for 5 min. If a mouse climbed on the maze walls, it was immediately returned to the abandoned arm. The start arm was varied between animals to avoid placement bias. An entry occurs when all four limbs of the mouse are within an arm. Re-entries into the same arm were rated as separate entries. An alternation is defined as consecutive entries into all three arms, and an overlapping technique was used. The number of arm entries and alternations were recorded to calculate the percentage of alternation behavior. The percent alternation was defined as consecutive entries in 3 different arms, divided by the number of possible alternations (total arm entries minus 2) times 100). A high percentage was an indication of good working memory as this indicated that the mouse recalled which arms it had already visited. Mice with less than 8 arm entries during the 5-min trial were excluded from the analysis.

### Motor function

Motor function was assessed for mice in all groups to see if there was any difference in the propensity of causing skeletal muscle weakness at 10 months of age with the rotarod (IITC Series 8, Life Sciences, Woodland Hills, CA), using a method we described previously[3, 6]. Mice were brought to the testing room at least 1 hour before the test to get acclimated to the environment. Two 60 s training trials at a constant speed of 9 rpm were performed with a 30-minute interval between trials. Then three 120 s test trials with a gradually increasing speed (4–40rpm) were performed with 60 min intervals. The latency to fall from the rotarod was recorded and analyzed.

### Olfactory function

To determine any impact of the intranasal ERFR on olfaction, the animals’ ability to smell volatile odors and their natural tendency to use olfactory cues for foraging was measured, as we have described previously[6]. The 3-day testing protocol consisted of an odor familiarization exercise on day 1, food deprivation on day 2, and testing on day 3. On day 1, mice were placed in a clean cage containing 3 cm of fresh bedding and normal chow and water. Three “cookies” per cage were placed in the bedding and left overnight. Cages were inspected on the morning of day 2 to record the number of cookies consumed. Later on day 2, at approximately 4 pm, the normal mouse chow pellets were removed from the cages and the testing mice were fasted overnight, with free access to water during this time. The test was performed on day 3 at approximately 11 am, after 1 h acclimatization in the testing room. Mice were individually introduced into a clean cage containing clean bedding and allowed to acclimate to the cage for 5 min. In a second clean cage, a single cookie was buried beneath the bedding in a random corner of the cage and the mouse was placed in the second cage. The time necessary for the animal to retrieve the cookie with its front paws was manually recorded (latency) up to a maximum of 15 min.

### Statistical Analysis

Dantrolene brain and blood concentrations were measured and reported as mean ± 95% CI (confidence intervals) and were analyzed by the Mann Whitney test. Chest cavity and cardiac plasma concentrations were combined in statistical analyses. The data for the behavioral studies were presented as mean ± 95% CI and analyzed using one-way or 2-way ANOVA followed by the Tukey’s or Šídák’s multiple comparisons tests, as described in the figure legends. The significance level for all analyses was set at 95% (*P* < 0.05). GraphPad Prism 9 (GraphPad Software Inc.) was used to conduct all statistical analyses. Data are available in the Harvard Dataverse online data repository (https://dataverse.harvard.edu/dataset.xhtml?persistentId=doi:10.7910/DVN/MEWJ42).

## RESULTS

### Dantrolene brain/blood concentration ratio study

**Figure 1:**
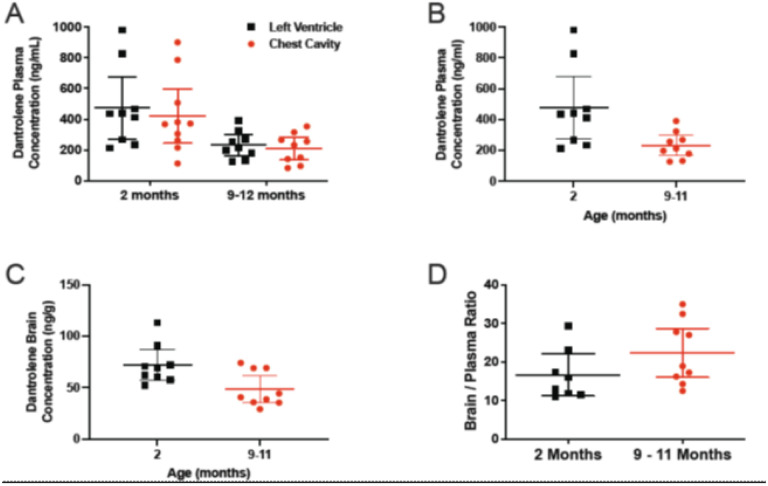
Comparison of the dantrolene concentrations in blood and brain with age. A) There was a significant decrease in the dantrolene plasma concentration from 2 to 9–11 months of age (p=0.001). There was no statistical difference in the dantrolene concentration found in the plasma from the left ventricle compared to the chest cavity at 2 months of age (p=0.793) nor at 9–11 months of age (p=0.966). At 2 months, n= 9 (left ventricle), n=9 (chest cavity), 9–11 months, n=9 (left ventricle and chest cavity). Data were analyzed by two-way ANOVA, with Šídák’s multiple comparisons test. B)The concentration of dantrolene in the plasma was significantly greater at 2 months compared with 9–11 months of age (p=0.008). C) Likewise, the dantrolene concentration in the brain was significantly greater at 2 months compared with 9–11 months of age (p=0.04). Data for B) and C) were analyzed with the Mann Whitney test, n=9 for all groups. D) The brain to plasma ratios at 2 months compared to 9–11 months were not significantly different (p=0.114). Data were analyzed with the Mann Whitney test, n=9 (2 months) and n=9 (9–11 months of age). All Data in the figure were expressed as the mean ± 95% CI.

The dantrolene concentration in the brain and blood was measured after a single intranasal exposure in young adult and aged mice. Blood samples were obtained from both the heart and chest cavity prior to euthanasia. We found that there was no significant difference in the dantrolene concentration found in the plasma from the left ventricle compared to the chest cavity at 2 months of age (p = 0.793) nor at 9–11 months of age (p = 0.966) (Fig. 1A) and thus the mean dantrolene concentration in the blood per animal was used for all analyses. However, there was a significant decrease in the dantrolene plasma concentration from 2 to 9–11 months of age (p = 0.001) (Fig. 1A). In comparison to young adult mice, the dantrolene concentration in the plasma of aged mice was significantly lower (232 ng/ml,476 ng/ml, respectively), (p = 0.008) (Fig. 1B) while the dantrolene concentration in the brain was significantly greater at 2 months compared with 9–11 months of age (p = 0.04) (Fig. 1C). The ratio of the dantrolene concentration in the brain to the concentration in the blood was used to compare dantrolene’s penetration through the blood brain barrier at each age. The brain/blood dantrolene concentration ratio trended higher in aged mice (Fig. 1D, P = 0.114) primarily due to the significantly lower dantrolene concentrations in the plasma.

### Effects of intranasal ERFR on behavior Cognitive Function

**Figure 2:**
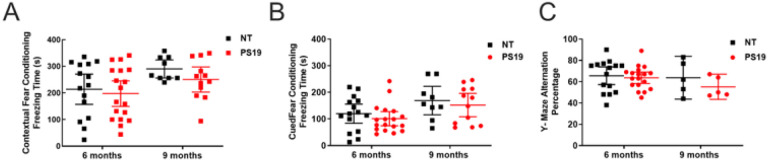
Baseline cognitive function in the PS19 tau transgenic mouse model with age. A) There was a significant difference in the contextual fear conditioning (FC) with age, with increased freezing times at 9 months compared to 6 months of age (adjusted p=0.012). There were no significant differences in freezing times between the PS19 mice and their non-transgenic (NT) littermates at either 6 months (adjusted p=0.836) or 9 months of age (adjusted p=0.522). For the NT mice, n=15 (6 months), n=9 (9 months); for PS19 mice n=18 (6 months), n=12 (9 months). B) There was also a significant difference in the cued FC test with age, with increased freezing times at 9 months compared to 6 months (adjusted p=0.007). There were no significant differences in freezing times between the PS19 mice and NT mice at either 6 months (adjusted p=0.639) or 9 months of age (adjusted p=0.797). NT, n=15 (6 months), n=9 (9 months); PS19 n=18 (6 months), n=12 (9 months). C) There was no significant difference with the Y-Maze with age (adjusted p=0.278). Likewise, there were no significant differences in alternation percentage between the PS19 mice and their non-transgenic littermates at either 6 months (adjusted p=0.892) or 9 months of age (adjusted p=0.508). NT, n=6 (6 months), n=5 (9 months); PS19 n=6 (6 months), n=5 (9 months). All data in the figure were expressed as the mean ± 95% CI and analyzed with a 2-way ANOVA, with Šídák’s multiple comparisons test.

Baseline cognitive function was first evaluated in tau transgenic PS19 mice and non-transgenic littermates at 9 and 11 months of age in the absence of intranasal ERFR. Interestingly, with the hippocampal-dependent contextual fear condition test, there was a significant difference with age, with increased freezing times at 9 months compared to 6 months of age (adjusted p = 0.012) (Fig. 2A). There were no significant differences in freezing times between the PS19 mice and their non-transgenic littermates at either 6 months (adjusted p = 0.836) or 9 months of age (adjusted p = 0.522). There was also a significant difference in the cued fear conditioning test with age, with increased freezing times at 9 months compared to 6 months (adjusted p = 0.007) (Fig. 2B). There were no significant differences in freezing times between the PS19 mice and their non-transgenic littermates at either 6 months (adjusted p = 0. 639) or 9 months of age (adjusted p = 0.797). Furthermore, there was no significant difference with the Y-Maze with age (adjusted p = 0.278). Likewise, there were no significant differences in alternation percentage between the PS19 mice and their non-transgenic littermates at either 6 months (adjusted p = 0.892) or 9 months of age (adjusted p = 0.508) (Fig. 2C).

**Figure 3:**
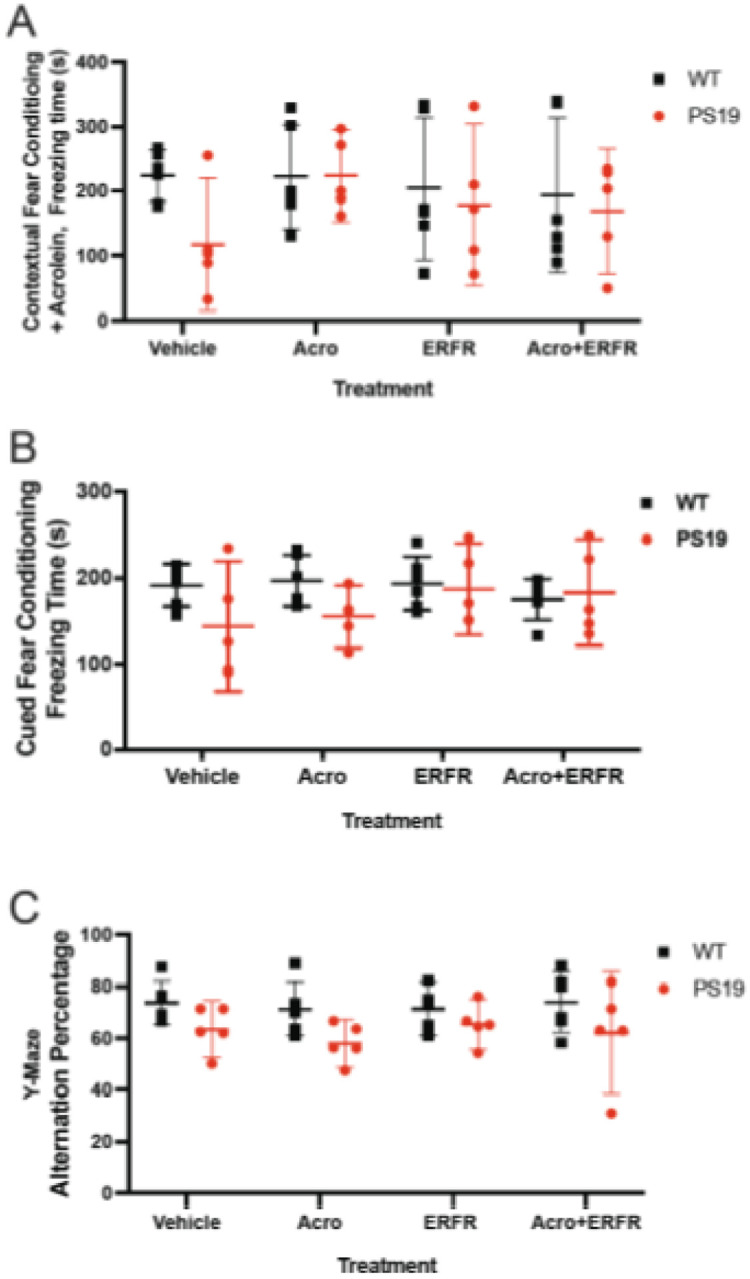
Cognitive function after intranasal ERFR and acrolein treatment in the PS19 tau transgenic mouse model. PS19 mice and wild type B6J mice (WT) were given 3 months - of intranasal dantrolene (ERFR), with or without acrolein treatments, and controls were given vehicle (water) or acrolein alone. A) For the hippocampal-dependent contextual fear conditioning test, there was no significant interaction between treatment and genotype (p=0.502). There were no significant differences in the freezing times between the WT and PS19 mice with any treatment (vehicle, adjusted p=0.474; acrolein, adjusted p>0.999; ERFR, adjusted p=0.999; ERFR+Acrolein, adjusted p>0.999). Furthermore, there were no significant differences between the PS19 ERFR treated mice compared to PS19 vehicle controls (adjusted p>0.999), or compared to the PS19 ERFR + acrolein treated mice (adjusted p>0.999). There was no significant difference between the PS19 ERFR+Acrolein treated mice and PS19 Acrolein control treated mice (p=0.969). In addition, there was no significant difference between the WT ERFR treated mice and WT vehicle treated control mice (p>0.999). For WT mice, n=6 and for PS19 mice, n=5 for all groups. B) For the cued, hippocampal-independent fear conditioning, there was no interaction between treatment and genotype (p=0.244). There were no significant differences in freezing times between the WT and PS19 mice with any treatment (vehicle, adjusted p=0.421; acrolein, adjusted p=0.590; ERFR, adjusted p>0.999; ERFR+Acrolein, adjusted p>0.999). Furthermore, there were no significant differences between the PS19 ERFR treated mice compared to PS19 vehicle controls (adjusted p=0.595), or compared to the PS19 ERFR + acrolein treated mice (adjusted p>0.999). There was no significant difference between the PS19 ERFR+Acrolein treated mice and PS19 Acrolein control treated mice (p=0.927). In addition, there was no significant difference between the WT ERFR treated mice and WT vehicle treated control mice (p>0.999). For WT mice, n=6 and for PS19 mice, n=5 for all groups. C) For the y-maze, there was an overall significant difference between the WT mice and the PS19 mice (p=0.003). However, the post-hoc tests indicated that there were no significant differences in the alternation percentage between the WT and PS19 mice for any treatment ((vehicle, adjusted p=0.746; acrolein, adjusted p=0.450; ERFR, adjusted p=0.985; ERFR+Acrolein, adjusted p=.602.). Furthermore, there were no significant differences between the PS19 ERFR treated mice compared to PS19 vehicle controls (adjusted p>0.999), or compared to the PS19 ERFR+acrolein treated mice (adjusted p>0.999). There was also no significant difference between the PS19 ERFR+Acrolein treated mice and PS19 Acrolein control treated mice (p=0.241). In addition, there was no significant difference between the WT ERFR treated mice and WT vehicle treated control mice (p>0.999). For WT mice, n=6 (vehicle, ERFR, ERFR+Acrolein), n=5 (Acrolein); for PS19 mice, n=5 for all groups. All data in the figure were expressed as the mean ± 95% CI and were analyzed using 2-way ANOVA with Tukey’s Multiple Comparisons test.

Acrolein treatment has been suggested as a novel animal model of sporadic AD (Zhang et al, 2020). Rats treated with acrolein daily for 8 weeks developed cognitive impairment (Huang et al 2013). Thus, in an attempt to accelerate cognitive impairment in the PS19 tau transgenic mouse model, acrolein was given to PS19 mice and non-transgenic littermates starting at 2 months of age, in conjunction with intranasal dantrolene (ERFR), and cognitive function assessed starting at 2 months of age. For the hippocampal-dependent contextual fear conditioning test, there was no significant interaction between treatment and genotype (p = 0.502). There were no significant differences in the freezing times between the WT and PS19 mice with any treatment (vehicle, adjusted p = 0.474; acrolein, adjusted p > 0.999; ERFR, adjusted p = 0.999; ERFR + Acrolein, adjusted p > 0.999). Furthermore, there were no significant differences between the PS19 ERFR treated mice compared to PS19 vehicle controls (adjusted p > 0.999), or compared to the PS19 ERFR + acrolein treated mice (adjusted p > 0.999). There was no significant difference between the PS19 ERFR + Acrolein treated mice and PS19 Acrolein control treated mice (p = 0.969). In addition, there was no significant difference between the WT ERFR treated mice and WT vehicle treated control mice (p > 0.999). (Fig. 3A). Similarly, for the cued fear conditioning test, hippocampal-independent fear conditioning, there was no interaction between treatment and genotype (p = 0.244). There were no significant differences in freezing times between the WT and PS19 mice with any treatment (vehicle, adjusted p = 0.421; acrolein, adjusted p = 0.590; ERFR, adjusted p > 0.999; ERFR + Acrolein, adjusted p > 0.999). Furthermore, there were no significant differences between the PS19 ERFR treated mice compared to PS19 vehicle controls (adjusted p = 0.595), or compared to the PS19 ERFR + acrolein treated mice (adjusted p > 0.999). There was no significant difference between the PS19 ERFR + Acrolein treated mice and PS19 Acrolein control treated mice (p = 0.927). In addition, there was no significant difference between the WT ERFR treated mice and WT vehicle treated control mice (p > 0.999)) (Fig. 3B). For the y-maze, there was an overall significant difference between the WT mice and the PS19 mice (p = 0.003). However, the post-hoc tests indicated that there were no significant differences in the alternation percentage between the WT and PS19 mice for any treatment ((vehicle, adjusted p = 0.746; acrolein, adjusted p = 0.450; ERFR, adjusted p = 0.985; ERFR + Acrolein, adjusted p = .602.). Furthermore, there were no significant differences between the PS19 ERFR treated mice compared to PS19 vehicle controls (adjusted p > 0.999), or compared to the PS19 ERFR + Acrolein treated mice (adjusted p > 0.999). There was also no significant difference between the PS19 ERFR + Acrolein treated mice and PS19 Acrolein control treated mice (p = 0.241). In addition, there was no significant difference between the WT ERFR treated mice and WT vehicle treated control mice (p > 0.999) (Fig. 3C).

#### Side effects of intranasal administration of ERFR and acrolein.

Overall, mice were observed to have good general health as evidenced by being well groomed and appropriate food intake and social behavior.

### Olfaction

**Figure 4:**
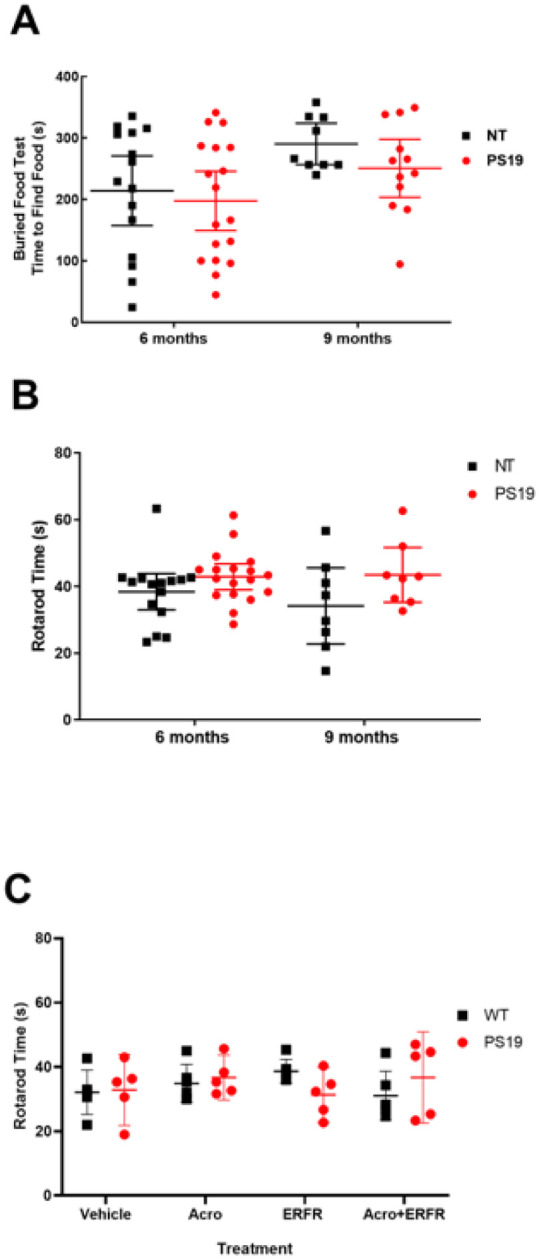
Potential side effects of chronic intranasal administration examined in PS19 tau transgenic mice. A) There was an overall significant effect of age with olfaction with the older mice taking longer to find the buried food (p=0.012). However, there were no effects of the intranasal ERFR treatment in PS19 mice compared to untreated non-transgenic littermates (NT) at either 6 months (adjusted p=0.836) or 9 months of age (adjusted p=0.522). For PS19 mice, n=18 (6 months) and n=12 (9 months) and for NT controls, n=15 (6 months) and n=9 (9 months). Data were analyzed using 2-way ANOVA with Šídák’s multiple comparisons test. B) No significant difference was found on rotarod performance between NT controls and PS19 mice treated with ERFR for 3 months at either 6 months (adjusted p value = (0.359) or at 9 months (adjusted p=0.128). For PS19 mice, n=18 (6 months) and n=8 (9 months) and for NT controls, n=15 (6 months) and n=8 (9 months).. Data were analyzed using 2-way ANOVA with Šídák’s multiple comparisons test. C) No significant differences in motor function were found between groups of WT ( or PS19 mice treated with ERFR and/or acrolein for 3 months (p=0.211). For NT and PS19 mice, n=6 for all groups. Data were analyzed using 2-way ANOVA. All data in the figure are expressed as the mean ± 95% CI.

There was an overall significant effect of age on olfaction, with the older mice taking longer to find the buried food (p = 0.012). However, there were no effects on olfactory function after intranasal ERFR treatment in PS19 mice compared to untreated non-transgenic littermates (NT) at either 6 months (adjusted p = 0.836) or 9 months of age (adjusted p = 0.522) (Fig. 4A).

### Motor function

Rotarod is a standard test to measure motor function in mice. After 3 months of treatment with ERFR, no significant difference was found on rotarod performance at 6 or 9 months of age, between the NT controls and PS19 mice (p = 0.195) (Fig. 4B). Likewise, in PS19 and wild type (B6J) mice treated for 3 months with intranasal dantrolene (ERFR), with or without acrolein, there were no significant interaction between the groups (p = 0.211) in rotarod performance (Fig. 4C).

## DISCUSSION

Considering the need for the chronic use of therapeutics for AD patients, the relatively higher dantrolene concentration in the CNS compared to the blood in the aged tau transgenic mice strengthens its therapeutic efficacy. Further understanding of the pharmacokinetics of dantrolene in the brain and peripheral blood in animals at different ages is important for the therapy design of dantrolene as a potential treatment for AD.

Our previous studies in mice clearly demonstrated a higher and prolonged dantrolene concentration in the brain with intranasal administration compared to oral use [6, 23]. Additionally, the intranasal dantrolene nanoparticle formulation provided better neuroprotection against cognitive dysfunction compared to the subcutaneous approach in 5XFAD mice. Furthermore, no significant side effects were detected in the nasal mucosa, or with olfactory, motor, or liver function, or morbidity and mortality, with up to 10 months intranasal administration[6, 29]. In this current collaborative research work, we used a dantrolene formulation, ERFR, created by Eagle Pharmaceuticals, Inc., to study a potential intranasal formulation of dantrolene for administration in future clinical studies. In comparison to our previous studies in 2 month old mice[6, 23], the intranasal administration of ERFR achieved significantly less brain or blood dantrolene concentrations at 20 minutes after administration, with a higher brain/blood dantrolene concentration ratio, though not significant. One difference with our previous studies is the technique used to measure the brain and blood dantrolene concentrations, which may contribute to some of the differences [6, 23]. The current study demonstrated that the intranasal administration of ERFR achieved significantly lower blood dantrolene concentrations in aged mice compared to young adult mice, possible due to decreased blood vessel number and supply to the nasal mucosa in aged mice. However, though the brain dantrolene concentration was significantly less in aged mice compared to that in young adult mice, the relative brain to blood ratio was higher, indicating a greater concentration of dantrolene in the brain. One possible mechanism is that the BBB in aged mice, and in AD patients, is disrupted and allows more uptake of dantrolene which typically has limited penetration into the CNS[30]. Nevertheless, this study suggests that intranasal administration of ERFR may provide adequate neuroprotective effects, with minimal systemic side effects.

The lack of a sporadic Alzheimer disease animal models is challenging for the Alzheimer field and a limitation for the development of treatments. Drugs that have proven effective in animal studies have not translated to the effective treatment of AD patients, 95% of whom have sporadic Alzheimer’s disease [1]. Although dantrolene has been shown to be neuroprotective in multiple familial Alzheimer disease animal models[3, 5, 6, 8, 31], its effects on a tauopathy animal model have not been tested previously. Neuronal tau accumulation is one of the hallmarks of Alzheimer’s disease and thought to contribute to brain atrophy and neurodegeneration. Among currently available tau pathology animal models, P301S mice (PS19) have been widely used for mechanistic studies and cognitive dysfunction has been reported at 5–6 months of age[25, 26, 32, 33]. Therefore, we hypothesized that intranasal dantrolene administration would ameliorate cognitive dysfunction in the PS19 tau transgenic mouse model.

Inconsistent with a previous study using this model[26] with reported cognitive dysfunction and motor deficits at around 5–6 months of age, the PS19 mice in our study did not demonstrate significant cognitive impairment or impaired motor function even at 9 months of age. It is possible that genetic drift is occurring, which has been reported in the lab that developed the model[25]. Although acrolein has been used to establish cognitive dysfunction similar to AD in WT rodents[27], the current study did not demonstrate acrolein’s neurotoxic effects to worsen cognitive function in either WT or PS19 transgenic mice. Thus, we could not determine whether or not intranasal ERFR was protective against cognitive dysfunction. Our study suggested that PS19 mice may not be a robust or reliable AD animal model to assess cognitive function going forward, and the therapeutic effectiveness of new or repurposed drugs for the treatment of AD.

Consistent with our previous study using a similar intranasal dantrolene nanoparticle formulation chronically[6], intranasal ERFR for up to 3 months did not affect body weight and general health, nor olfactory and motor function. So, intranasal ERFR may be relatively safe for chronic use, an initial but important step for the development of intranasal ERFR as a potential drug for the treatment of AD in patients.

This study has the following limitations: 1) This is a small-scale brain concentration study with a relatively small sample size and is thus prone to false negative results, though the sample size for the behavioral studies is considered adequate, based on our previous studies[3, 6]. 2) Dantrolene concentrations were measured at a single time point, so the full brain/plasma pharmacokinetics of intranasal ERFR were not obtained. 3) Control groups, such as oral or subcutaneous dantrolene administration, were not included to compare the pharmacokinetics of intranasal with commonly used administration methods. 4) The extent of tau pathology was not examined in the PS19 mice due to the lack of cognitive dysfunction, even with acrolein treatment. 5) While an acrolein dose response study was not undertaken, we elected to use an effective dose from the literature[27], although a higher dose may cause cognitive dysfunction in PS19 mice.

In summary, our study suggests that the intranasal administration of ERFR provides a favorable dantrolene brain to blood concentration ratio in aged mice compared to young adult mice and had no serious systemic side effects with chronic use, suggesting its potential as a treatment for older AD patients. PS19 tau transgenic mice may not be a robust AD animal model to test the therapeutic efficacy of new or repurposed drugs on cognitive function in future studies.
